# Localization and Expression of Aquaporin 1 (AQP1) in the Tissues of the Spiny Dogfish (*Squalus acanthias*)

**DOI:** 10.3390/ijms26125593

**Published:** 2025-06-11

**Authors:** Christopher P. Cutler, Bryce MacIver

**Affiliations:** 1 Department of Biology, Georgia Southern University, Statesboro, GA 30460, USA; 2 Department of Medicine, Beth Israel Deaconess Medical Center, Harvard Medical School, Boston, MA 02115, USA; imaciver@bidmc.harvard.edu

**Keywords:** Aquaporin 1, spiny dogfish, osmoregulation, localization

## Abstract

Aquaporin 1 is a membrane water channel protein, which was studied here in spiny dogfish (*Squalus acanthias*) osmoregulatory tissues using a variety of techniques. The cloning of aquaporin 1 (*AQP1*) in the spiny dogfish identified a splice variant version of the mRNA/protein (*AQP1SV1*/AQP1SV1). Polymerase chain reaction (PCR) in a range of tissues showed *AQP1* to be expressed at very high levels in the rectal gland with ubiquitous mRNA expression at lower levels in other tissues. Northern blotting showed that *AQP1* had a mRNA size of 5.3 kb in kidney total RNA. The level of *AQP1* mRNA was significantly lower in the rectal glands of fish acclimated to 120% seawater (SW; vs. 75% SW (*p* = 0.0007) and 100% SW (*p* = 0.0025)) but was significantly higher in those fish in the kidney (vs. 100% SW (*p* = 0.0178)) and intestine (vs. 75% SW (*p*= 0.0355) and 100% SW (*p* = 0.0285)). Quantitative PCR determined that *AQP1SV1* mRNA levels were also significantly lower in the rectal glands of both 120% (*p* = 0.0134) and 100% SW (*p* = 0.0343) fish in comparison to 75% SW-acclimated dogfish. Functional expression in *Xenopus* oocytes showed that AQP1 exhibited significant apparent membrane water permeability (*p* = 0.000008–0.0158) across a range of pH values, whereas AQP1SV1 showed no similar permeability. Polyclonal antibodies produced against AQP1 (AQP1 and AQP1/2 antibodies) and AQP1SV1 had bands at the expected sizes of 28 kDa and 24 kDa, respectively, as well as some other banding. The weak AQP1 antibody and the stronger AQP1/2 antibody exhibited staining in the apical membranes of rectal gland secretory tubules, particularly towards the periphery of the gland. In the gill, the AQP1/2 antibody in particular showed staining in secondary-lamellar pavement-cell basal membranes, and in blood vessels and connective tissue in the gill arch. In the spiral valve intestine side wall and valve flap, the AQP1/2 antibody stained muscle tissue and blood vessel walls and, after tyramide signal amplification, showed some staining in the apical membranes of epithelial cells at the ends of the luminal surface of epithelial folds. In the rectum/colon, there was also some muscle and blood vessel staining, but the AQP1 and AQP1/2 antibodies both stained a layer of cells at the base of the surface epithelium. In the kidney convoluted bundle zone, all three antibodies stained bundle sheath membranes to variable extents, and the AQP1/2 antibody also showed staining in the straight bundle zone bundle sheath. In the kidney sinus zone, the AQP1/2 antibody stained the apical membranes of late distal tubule (LDT) nephron loop cells most strongly, with the strongest staining in the middle of the LDT loop and in patches towards the start of the LDT loop. There was also a somewhat less strong staining of segments of the first sinus zone nephron loop, particularly in the intermediate I (IS-I) tubule segment. Some tubules appeared to show no or only low levels of staining. The results suggest that AQP1 plays a role in rectal gland fluid secretion, kidney fluid reabsorption and gill pavement-cell volume regulation and probably a minor role in intestinal/rectal/colon fluid absorption.

## 1. Introduction

Aquaporins (AQPs) are a family of water and small solute membrane channel proteins, and in mammals, the family contains thirteen members (AQP0-12) [1,2]. AQP1 was the first aquaporin identified by Peter Agre’s lab in 1991, for which he received the Nobel Prize in chemistry in 2003 [3]. Mammalian aquaporin water channels fall into three rough groups: water-selective AQPs (AQP0, -1, -2, -4, -5 and -6); aquaglyceroporins (AQP3, -7, -9 and -10), which are additionally permeable to small solutes such as glycerol and urea; and more divergent AQPs (AQP8, -11 and -12) [1]. In wider vertebrate species there are also an additional four known AQPs (AQPs 13–16) [4]. In the elasmobranch genome there are now known to be a complement of thirteen *AQP*s (*AQP0*, *AQP1*, two copies of *AQP3*, *AQP4*, *AQP8*, *AQP9*, two copies of *AQP10*, *AQP11*, *AQP12*, *AQP14* and *AQP15*) [4,5,6]. However, *AQP1* was first identified in elasmobranchs when very little was known about them in sharks. The initial elasmobranch AQP identified was *AQP15* (then called *AQP1e*) in the bull shark (*Carcharhinus leucas*) [7]. Attempts were then made to amplify homologs of *AQP15* from spiny dogfish cDNA by using a degenerative PCR approach (see [6] for example), but these attempts instead resulted in the amplification of dogfish *AQP1*, with dogfish *AQP15* being quickly identified afterwards [8,9]. The reason for this is that *AQP1* and *AQP15* have a level of similarity because they both result from an ancient duplication event and hence share a relatively high level of homology [4].

The distribution of AQP1 expression in mammals has been shown to be fairly ubiquitous, with most tissues, except the testes and leukocytes, showing some *AQP1* expression [1]. Similarly, in the dogfish, initial studies using an exhaustive PCR approach (of a small high-amplification-efficiency fragment using a high number of {35} cycles), amplified *AQP1* from every one of the ten tissue cDNAs studied [10].

Elasmobranchs such as the spiny dogfish have a different osmoregulatory approach in comparison with mammals or teleost fish [10]. Marine elasmobranchs have body fluids of a similar osmolality (slightly hyperosmotic) to the seawater (SW) environment most elasmobranch species live in [11,12,13]. They achieve this by both retaining additional NaCl in comparison to teleost fish but also by retaining and even producing extra urea [11,14,15]. Due to the slightly higher plasma osmolality and also lower salt levels than the environmental milieu, there is a persistent slight net influx of water and salt across the gills and, despite very low brachial urea permeability, a loss of urea [14,16,17,18,19]. Salts and water are also absorbed both from a small amount of SW imbibed but also from that ingested during feeding and from the food itself [13]. Elasmobranchs also have a rectal gland attached to the side of the colon/rectum at the end of the gastrointestinal tract. The rectal gland secretes an approximately iso-osmotic NaCl solution which has a very low level of urea (7–28.8 mmol/L) [11,14,20]. The rectal gland is highly regulated to respond to elevated body fluid volume levels and is principally engaged in excreting excess fluid. That is, it essentially eradicates water, which fluid is largely composed of but for which there are no active transport systems available to move directly; hence, solutes (NaCl) have to be secreted to get the water to follow by osmosis [21,22,23].

An essential component of the elasmobranch osmoregulatory strategy is the kidney, which engages in high levels of reabsorption of urea and another solute, TMAO (trimethylamine oxide), which counteracts the denaturing effects of high levels of plasma urea [14,17]. This is such that only 10–15% of urea from the glomerular filtrate is excreted [14,17]. Additionally, and possibly to facilitate urea reabsorption, 60–70% of filtered NaCl and 60–85% of filtered fluid (water) are reabsorbed. This, however, still results in urine that is hypo-osmotic compared to plasma by 50–250 mOsm/L [17,24,25]. The elasmobranch nephron is more complex than in mammals. The nephron runs through two zones, the bundle zone (straight and convoluted), surrounded by a bundle sheath, and the sinus zone, with blood sinus spaces between tubules. The nephron starts at the glomerulus just outside of the bundle zone. The tubule emerging from the glomerulus is the neck segment which forms a loop through the bundle zone, with the returning tubule of the loop being the proximal Ia (PIa) tubule. The tubule re-emerges into the sinus zone with a loop formed of the proximal Ib (PIb) tubule, followed by the proximal II (PII) and intermediate I (IS-I) nephron segments. The tubule then goes into a second bundle zone loop with the intermediate II segment (IS-II), which returns with the early distal tubule (EDT) segment. This then re-emerges into the sinus zone and becomes the late distal tubule (LDT) loop, before finally going back into the bundle zone as the collecting tubule (CT), which eventually exits the bundle zone as the collecting duct (CD). For a model of the nephron with this terminology, see [26]; but also see [27,28,29,30,31], which have variations in terminology. In cross-sections of the kidney, two parts of the bundle zone can often be seen: the convoluted lateral bundle zone, where bundles of the five tubules are often seen in cross-section, and the straight bundle zone, where tubules are running back and forth to the lateral bundle zone from the sinus zone and are seen in longitudinal section. A model concerning the locations of the reabsorption of salt (NaCl), water and urea originally hypothesized that NaCl was largely reabsorbed in the EDT, water was likely significantly reabsorbed in the LDT nephron loop in the sinus zone, with urea reabsorbed in the CT bundle zone segment [32]. However, it has also been suggested that some NaCl, water and urea reabsorption occurs in more proximal regions of the nephron (in the first sinus zone loop), where water would follow solute reabsorption by osmosis through either or both of the transcellular or paracellular pathways [16,33,34]. Indeed, in the spiny dogfish, the expression of the UT-1 urea transporter appears to occur largely in the sinus zone loops [35].

To date, information concerning a number of different dogfish AQPs in osmoregulatory tissues has been published. In the renal nephron, AQP4 and -15 are expressed at high levels at the start of the LDT with AQP3 expression being highest towards the end of the LDT and with AQP3-2 showing variable expression along the LDT [26,36,37]. These LDT AQPs, therefore, are consistent with major water absorption occurring in this nephron segment [32]. AQP4 also has lower levels of expression in the first sinus zone loop. AQP4 and AQP3-2 are also expressed in the bundle zone EDT segment, and AQP15 is expressed in the bundle sheath, where it probably provides water to allow the creation of fluid for the solutes being reabsorbed from bundle nephrons [26,37,38]. In the gill, AQP4 was shown to be expressed in two types of large mitochondrial-rich-like cells co-expressing Na,K-ATPase or V-type ATPase, and AQP15 was expressed in certain pavement cells of the secondary lamellae, although the function of these AQPs in the gill is unclear [36,38]. In the rectal gland, AQP4 was shown to be expressed in the basolateral membranes of the secretory tubules [36]. In the spiral valve intestine, AQP4 was shown to be expressed in the underlying muscle tissue [10]. AQP3 was shown to be expressed in the basal membranes of surface epithelial cells, whereas AQP15 was found in the apical and lateral membranes of those cells in both the side wall and spiral valve flap epithelia [38]. This suggests that AQP3 and AQP15 may be involved in water absorption in the intestine.

This study has sought to build on initial cloning work concerning spiny dogfish *AQP1* [8,9,10] and identify the locations and characteristics of the expression of this gene/protein in various osmoregulatory tissues. During the initial cloning work to obtain the complete *AQP1* coding sequence, an additional 3′ end sequence was also identified, called *AQP1SV1*, and this had a different C-terminal amino acid sequence compared with *AQP1* itself and was deemed to be a *AQP1* splicing variant. To determine if AQP1 plays a role in osmoregulatory processes, total RNA samples from fish acclimated to different environmental salinities (75% SW–120% SW) were examined for changes in *AQP1* expression in several osmoregulatory tissues. These salinities were chosen to stress the osmoregulatory system without leading to mortality of the fish.

This work presents the first information on the role of *AQP1*/AQP1 in elasmobranchs and as such is discovery science. However, as with the sequencing of the genomes of organisms, it is vital to have basic information to formulate hypotheses for further investigations. Hence, these kinds of studies are still necessary and indeed critical to the advancement of science in this area, where little or nothing is currently known. The results from this study suggest several avenues for future research, but the most obvious would concern investigations into the role of AQP1 in rectal gland fluid secretion and the control of *AQP1* expression by various factors known to regulate rectal gland fluid secretion.

## 2. Results

**Figure 1:**
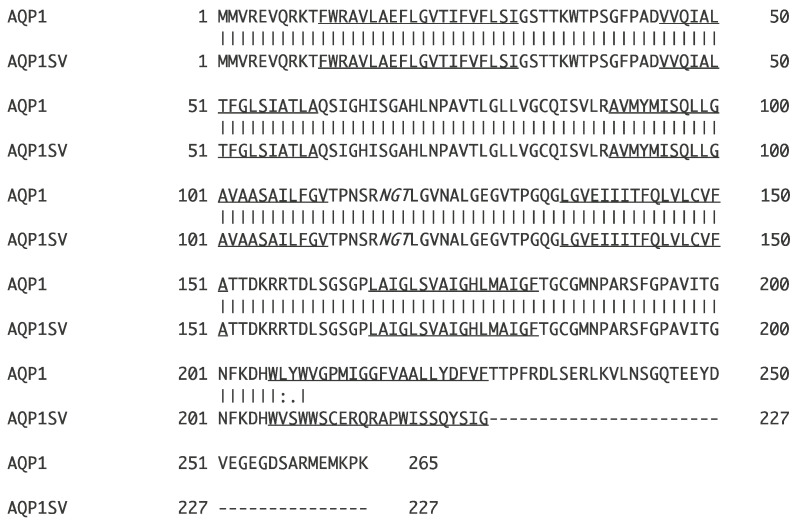
Derived amino acid sequences of AQP1 and AQP1SV1. A potential N-glycosylation site in the center of the sequence is in italics; this would potentially add to the molecular weight of the protein if this residue were glycosylated. Potential core hydrophobic membrane-spanning regions are underlined.

The original experimental primers used to identify what is now *AQP15* (then called *AQP1e*) in the spiny dogfish were based on the AQP15 sequence from the bull shark (*Carcharhinus leucas* [7]), but instead of amplifying *AQP15*, they amplified a fragment of *AQP1* from rectal gland cDNA [8,9]. During RACE PCR experiments to complete the cDNA sequence of *AQP1*, a second sequence was obtained from the 3′ RACE experiments using rectal gland marathon cDNA. The two transcripts are here referred to as *AQP1* and *AQP1SV1* (see Figure 1). AQP1SV1 had a different C-terminal amino acid sequence.

**Figure 2:**
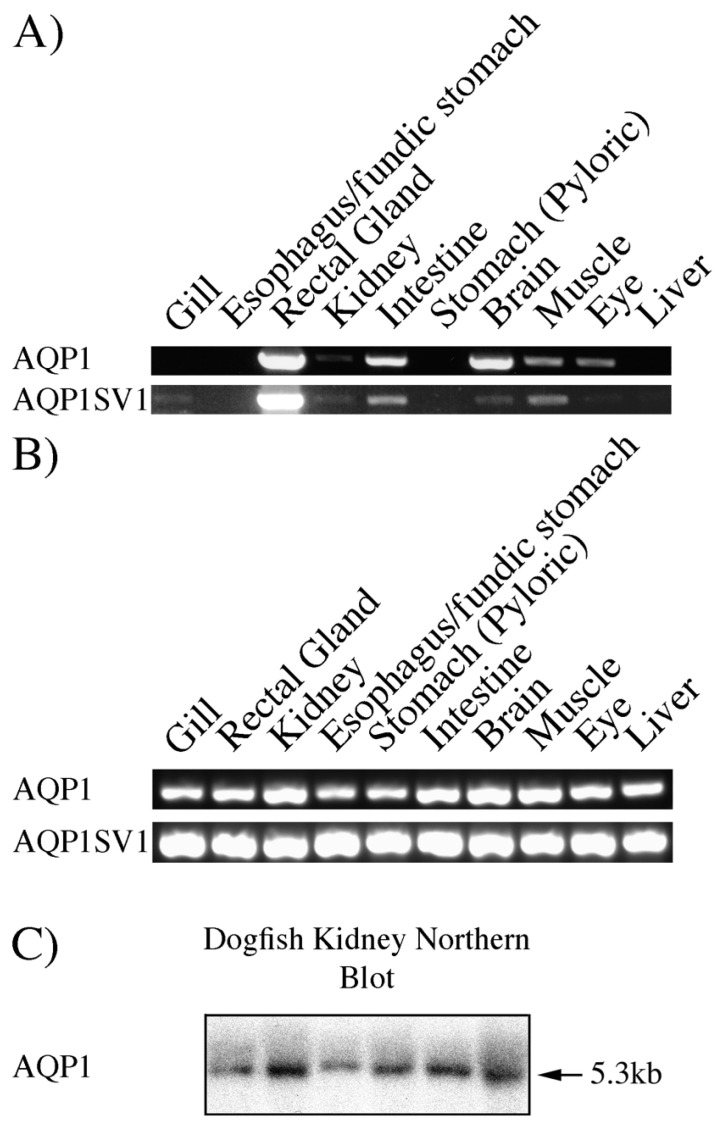
Tissue PCRs (**A**,**B**) and Northern blotting (**C**) using spiny dogfish total RNAs/cDNAs. (**A**) PCR reactions amplifying cDNAs for the whole coding region of *AQP1* and its splice variant *AQP1SV1*. PCR reactions were carried out using 30 cycles. (**B**) More efficient reactions, amplifying a short fragment using 35 cycles and a more efficient polymerase. The image for *AQP1* that previously appeared in [10]. (**C**) Northern blot using the short *AQP1* PCR fragment as a probe and using six 2.5 μg samples of kidney total RNA. Agarose gel electrophoresis/RNA size markers determined the *AQP1* mRNA’s size to be 5.3 kb.

Initial experiments looked at the tissue distribution of the splice variants using PCR in 10 tissue cDNAs already available (see Figure 2A). This showed a fairly similar distribution of mRNA expression for both *AQP1* and the splice variant version *AQP1SV1*. They both showed extremely high levels of expression in the rectal gland, with lower levels in the brain and intestine and with very low levels in the gill, kidney, muscle, eye and liver. Further, more exhaustive tissue PCR experiments (Figure 2B), designed to give signals from lower levels of mRNA/cDNA, showed expression in every one of the tissues studied.

**Figure 3:**
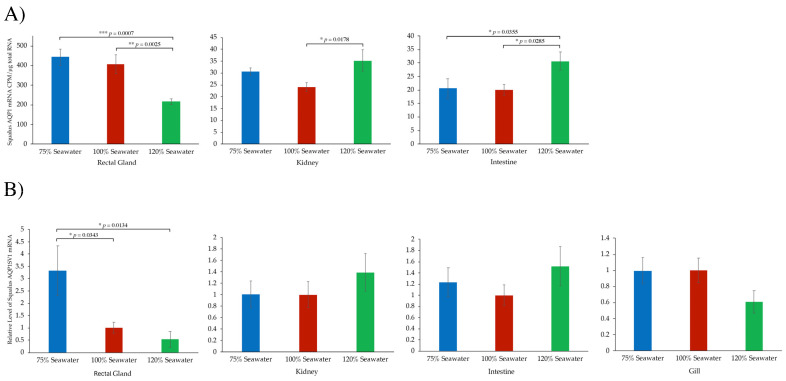
(**A**) Quantification of radioactive (^32^P dCTP) signals on Northern blots using an *AQP1*-specific probe. Bands on the Northern blot were quantified using Instant Imager. (**B**) QPCR of *AQP1SV1* mRNA levels in four tissues using *AQP1SV1* QPCR primers (see Table 1). Abundance levels are relative to the level in 100% SW fish. There were *n* = 6 fish per experimental group. Graphs have SEM error bars. Statistical testing used ANOVA statistics with Fisher’s PLSD post hoc test. * *p* < 0.05, ** *p* < 0.01 and *** *p* < 0.001.

Northern blotting and QPCR was also carried out using total RNA samples from an acclimation experiment where six spiny dogfish were acclimated to either 75% or 120% of the local (100%) SW (see Figure 2C and Figure 3).

For *AQP1*, again, the rectal gland showed the highest levels of mRNA expression with a roughly 16-fold higher level than that seen in the kidney and intestine (Figure 3A). In rectal gland *AQP1*, mRNA levels were significantly lower in 120% SW fish than in either 100% SW or 75% SW dogfish. In the kidney there was a significantly higher *AQP1* mRNA level in 120% SW compared with 100% SW dogfish, whereas in the intestine, the 120% SW dogfish had significantly higher *AQP1* mRNA levels than either 100% or 75% SW dogfish. With the splice variant (AQP1SV1; Figure 3B), mRNA levels were only significantly different in the rectal gland, where the 75% SW fish had significant higher mRNA levels than the 100% SW and 120% SW dogfish.

**Figure 4:**
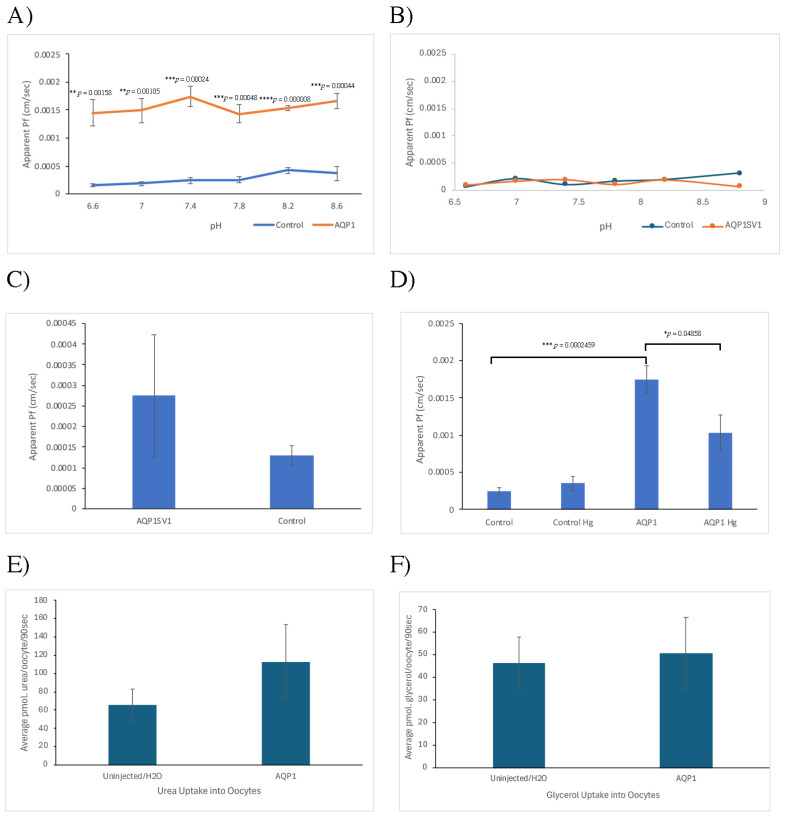
Functional expression of *AQP1* and *AQP1SV1* in *Xenopus* oocytes. (**A**) Comparison of apparent water permeability (Pf) of oocytes expressing AQP1 compared with water-injected control oocytes across a range of pH values. Means are of 4 experiments. (**B**) Comparison of apparent water permeability (Pf) of oocytes expressing AQP1SV1 compared with water-injected control oocytes, across a range of pH values, in a single experiment. (**C**) Comparison of apparent water permeability (Pf) of oocytes (at pH 7.4), expressing AQP1SV1 compared with un-injected or water-injected control oocytes. Means are of 3 experiments. (**D**) The effect of mercury (Hg) on the apparent water permeability (Pf) of oocytes expressing AQP1 compared with water-injected control oocytes. Means are from 3 experiments using mercury (not significantly different) and 4 non-mercury experiments. (**E**) The uptake of urea into AQP1-expressing oocytes compared with un-injected or water-injected control oocytes. Mean are from 3 experiments. (**F**) The uptake of glycerol into AQP1-expressing oocytes compared with un-injected or water-injected control oocytes, Means are from 3 experiments. Graphs (panels (**A**,**C**–**F**)) have SEM error bars. Statistical significance was determined by Student’s T-test using Microsoft Excel. Statistical comparisons in panels (**C**,**E**,**F**) were not significantly different. * *p* < 0.05, ** *p* < 0.01, *** *p* < 0.001 and **** *p* < 0.0001.

The functional studies expressing *AQP1* and *AQP1SV1* in *Xenopus* oocytes showed that AQP1 has similar water permeability to spiny dogfish AQP4 and is also similarly unaffected by different pH values (Figure 4A; [39]). In conjunction with other species, AQP1 was also sensitive to non-specific inhibitor mercury (Figure 4D); although the decrease was not statistically significant), and AQP1 showed no real glycerol or urea permeability (Figure 4E,F; [40]). However, the splice variant version AQP1SV1 showed no sign of any significant water permeability at pH 7.4 (Figure 4C) or at any pH value tested in a single experiment (Figure 4B).

In functional expression studies using *Xenopus* oocytes (Figure 4), AQP1 showed significantly higher apparent water permeability compared with water-injected control oocytes across a range of pH values (Figure 4A). In similar experiments where oocytes were instead injected with *AQP1SV1* mRNA, there appeared to be no significant apparent water permeability (Figure 4B,C). The non-specific inhibitor of aquaporin function, mercury, reduced the level of apparent AQP1 water permeability, although the level of reduction was not statistically significant (Figure 4D). Studies investigating the uptake of urea or glycerol also showed no statistically significant elevated influx of these solutes in AQP1-expressing oocytes compared with un-injected control oocytes (Figure 4E,F).

**Figure 5:**
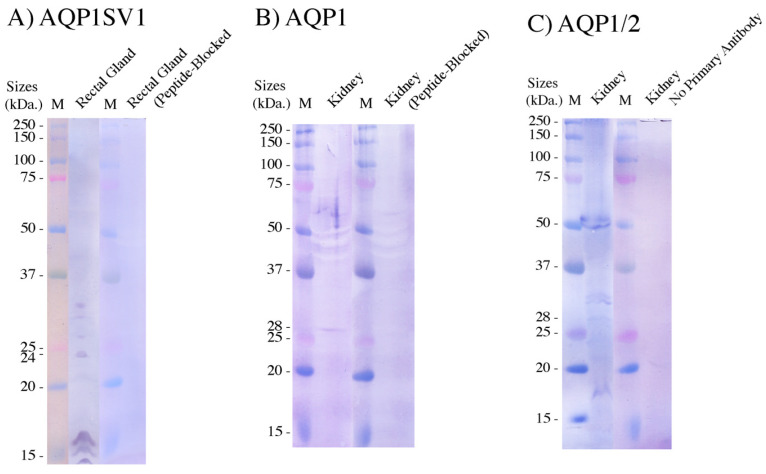
Western blots of 300 μg of (**A**) rectal gland crude membrane proteins and (**B**,**C**) kidney plasma membranes. (**A**) was incubated with the AQP1SV1 antibody for 1 h or the antibody was pre-blocked (1 h, 50 μg/mL peptide) with the peptide antigen. Blots were incubated in the alkaline phosphatase substrate NBT/BCIP for 5 min. Precision Plus Kaleidoscope molecular protein markers (M; Biorad, Santa Rosa, CA, USA) were run on an adjacent filter strip on the same electrophoresis gel. (**B**) was incubated with the AQP1 antibody (as in (**A**)), but strips were incubated for 12 min in NBT/BCIP reagent. (**C**) was incubated with the AQP1/2 antibody and with the NBT/BCIP reagent for 5 min. The control strip had the AQP1/2 antibody omitted (no primary antibody) from the procedure.

Once the nucleotide sequences of *AQP1* and its splice variants were available, two antibodies against the C-termini of AQP1 and AQP1SV1 were produced. Both antibodies only gave weak signals. The AQP1SV1 antibody gave a band of the expected size (24 kDa) on the Western blots of rectal gland crude membrane extracts (Figure 5A), but there were also three larger bands between 28 and 33 kDa and some low-molecular-weight banding around 15 kDa. None of these bands were seen on the corresponding peptide-antigen-blocked control antibody blot. For the AQP1 antibody, using kidney plasma membrane protein samples had an extremely long incubation time (12 min) in the alkaline phosphatase reagent (NBT/BCIP), which produced a very weak single band of an expected size of 28 kDa, which was not present on the corresponding control peptide-blocked antibody blot. With the subsequently produced AQP1 antibody (AQP1/2), stronger banding was seen around 28 kDa (produced with 5 min NBT/BCIP incubation) with two further bands being seen around 31 and 32 kDa. There were also a band around 250 kDa, two bands around 52 and 54 kDa and a low-molecular-weight band seen at 18 kDa. None of the bands from the AQP1/2 antibody were seen on the control blot.

**Figure 6:**
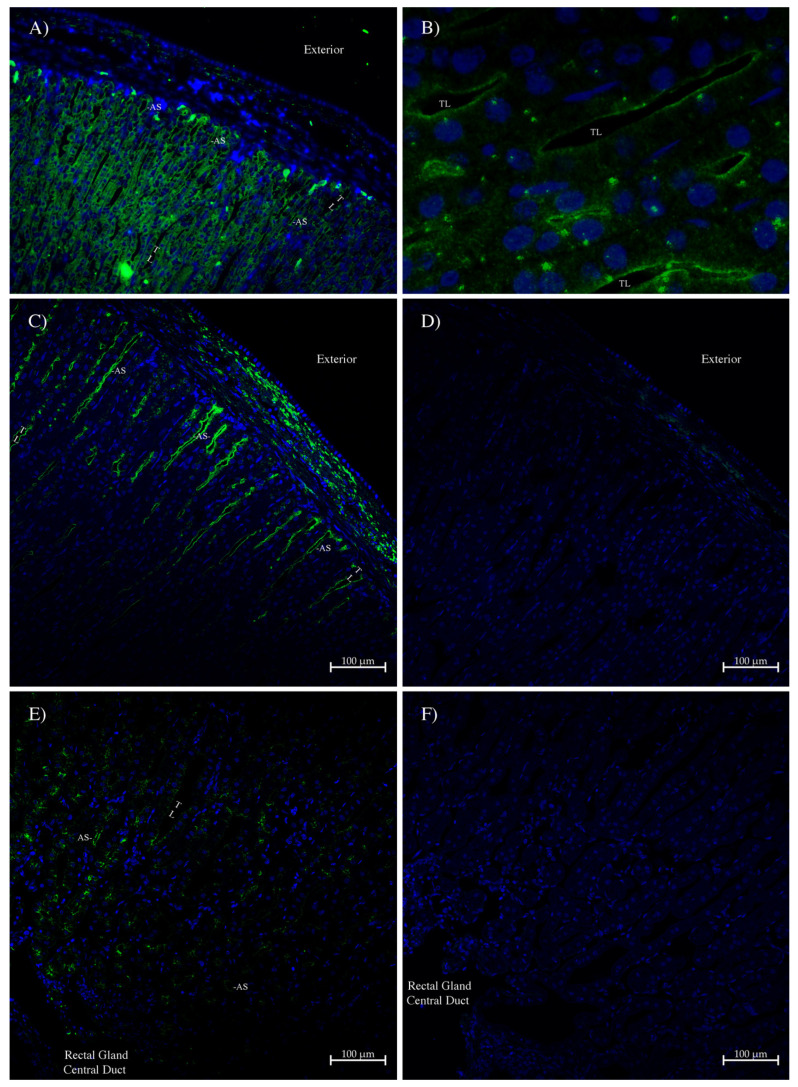
Immunohistochemistry of the spiny dogfish rectal gland. (**A**) at 100× magnification and (**B**) at 400× magnification were imaged with a Zeiss Axiovert microscope (Zeiss, Oberkochen, Germany). Green staining came from an Alexa 488 dye-labeled donkey anti-rabbit IgG secondary antibody (Thermofisher, Carlsbad, CA, USA). Images (**C**–**F**) were imaged on a Zeiss 710 laser-scanning confocal microscope by using a 20× lens, with a zoom setting of 0.6, with scale bars as shown. Antibody staining (green) was generated using a tyramide amplification kit (Thermofisher). Sections (**D**,**F**) are control serial sections that lack the primary AQP1/2 antibody. In all sections, the blue stain is DAPI nuclear counterstain. Sections (**C**–**F**) were processed with True Black autofluorescence quencher (Biotium, Fremont, CA, USA). Exterior = outside of the gland. AS = apical membrane staining. TL = tubule lumen. The rectal gland central duct is also indicated.

The antibodies were also used in immunohistochemistry to localize the proteins in various tissues of the spiny dogfish. In the rectal gland (Figure 6), some weak staining was seen with the AQP1 antibody in the apical membrane of secretory tubules around the outer edge of the gland (Figure 6A,B). Likewise, similar but much stronger staining was seen with the AQP1/2 antibody (Figure 6C). Staining was absent in the control that omitted the antibody (Figure 6D,F). Much lower levels of staining were seen in secretory tubules towards the central rectal gland duct (Figure 6E).

**Figure 7:**
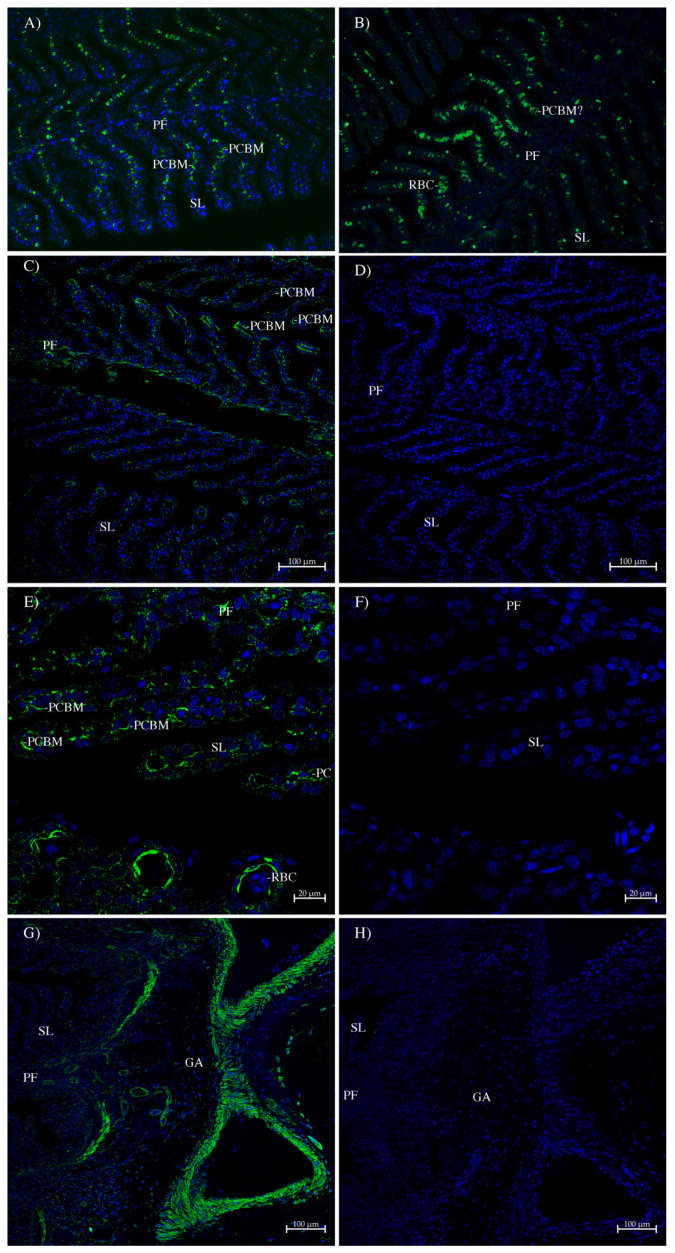
Immunohistochemistry of spiny dogfish gill. Sections (**A,B**) (100× magnification) were imaged with a Zeiss Axiovert microscope. Green staining comes from (**A**) the AQP1 antibody and (**B**) the AQP1SV1 antibody both incubated with an Alexa 488 dye-labeled donkey anti-rabbit IgG secondary antibody (Thermofisher). Sections (**C**–**H**) were imaged on a Zeiss 710 laser-scanning confocal microscope and utilized the AQP1/2 antibody detected by an Alexa 488 plus dye-labeled donkey anti-rabbit IgG secondary antibody (Green staining; Thermofisher). These sections were processed with True Black Plus autofluorescence quencher (Biotium). Images (**D**,**F**,**H**) are control serial sections (comparable to (**C**,**E**,**G**), respectively) incubated with peptide-antigen-blocked antibody (1 h, 50 μg/mL peptide). Images (**C**,**D**) were produced with a 20× lens and a zoom setting of 0.6. Images (**E**,**F**) were produced with a 40× lens and a zoom setting of 1.0, all with scale bars as shown. Images (**G**,**H**) were produced with a 10× lens and a zoom setting of 1.0. In all sections, the blue stain is DAPI nuclear counterstain. PF = primary filament; SL = secondary lamellae; GA = gill arch; RBC = red blood cell; PC = pillar cell; and PCBM = pavement-cell basal membrane.

In the gill, the AQP1 and AQP1SV1 antibodies (Figure 7A and 7B, respectively) seemed largely to be staining red blood cells. This may be because these images were taken before the availability of the autofluorescence quencher, True Black (Biotium). However, there were some small indications in places of potential pavement-cell basal membrane (PCBM) staining. As usual, the AQP1/2 antibody (Figure 7C–H) gave much stronger staining and clearly stained the basal membranes of pavement cells (Figure 7C,E); this is in comparison to control serial sections incubated with peptide-antigen-blocked antibody. Note also (in Figure 7E) that red blood cells do not show any staining. The AQP1/2 antibody also stained the smooth muscle/connective tissue surrounding blood vessels in the gill arch and at the base of primary filaments (Figure 7G).

**Figure 8:**
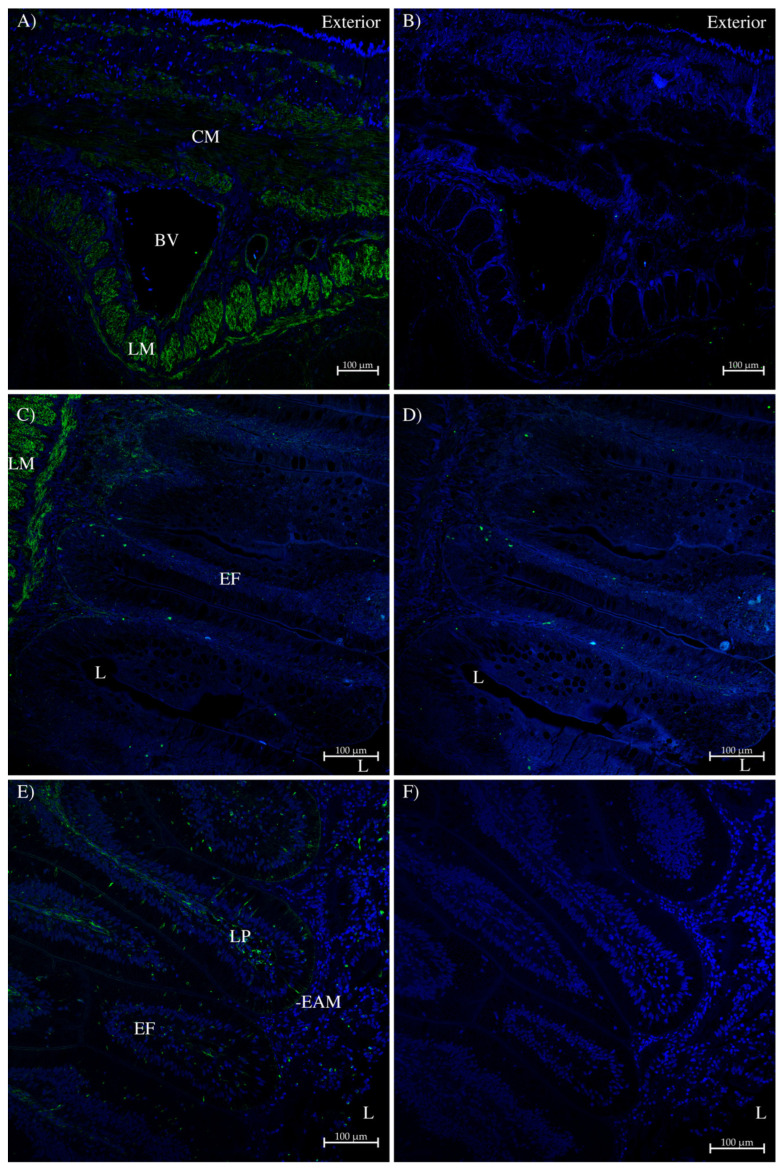
Immunohistochemistry of spiny dogfish spiral valve intestinal side wall. Sections were imaged on a Zeiss 710 laser-scanning confocal microscope. Sections (**A**,**B**) were imaged with a 10× lens with a zoom setting of 0.9. Sections (**C**–**F**) were imaged with a 20× lens with a zoom setting of 0.6. In sections (**A**,**C**), AQP1/2 antibodies were stained green with Alexa 488 plus dye-labeled donkey anti-rabbit IgG secondary antibody (Thermofisher). Sections (**B**,**D**) are control serial sections (comparable to (**A**,**C**), respectively) incubated with peptide-antigen-blocked antibody (1 h, 50 μg/mL peptide). Section (**E**) was generated using a tyramide amplification kit (Thermofisher). Section (**F**) is a control serial section that lacked the primary AQP1/2 antibody. CM = circumferential muscle; LM = longitudinal muscle; BV = blood vessel; L = intestinal lumen; EF = epithelial fold; LP = lamina propria; and EAM = epithelial apical membrane. All sections were processed with True Black autofluorescence quencher (Biotium), and the blue stain is DAPI nuclear counterstain. Sizes are indicated with scale bars.

In the side wall of the intestine, immunohistochemistry using the AQP1/2 antibody (Figure 8A,C) showed that AQP1 was expressed in circumferential and longitudinal muscle and in the smooth muscle/connective tissue surrounding blood vessels. There was only sporadic individual cell staining in the surface epithelial layer. Control serial sections incubated with peptide-antigen-blocked antibody (Figure 8B,D) showed no similar staining, except for the staining of a few individual cells in the surface epithelial layer. However, when the antibody staining was amplified using a tyramide amplification kit (Thermofisher; Figure 8E), some light staining was seen in the apical membranes of epithelial cells (EAM), as well as in the lamina propria (LP) in the middle of the epithelial folds (EFs). A control serial section (Figure 8F) with no AQP1/2 antibody showed no similar staining.

**Figure 9:**
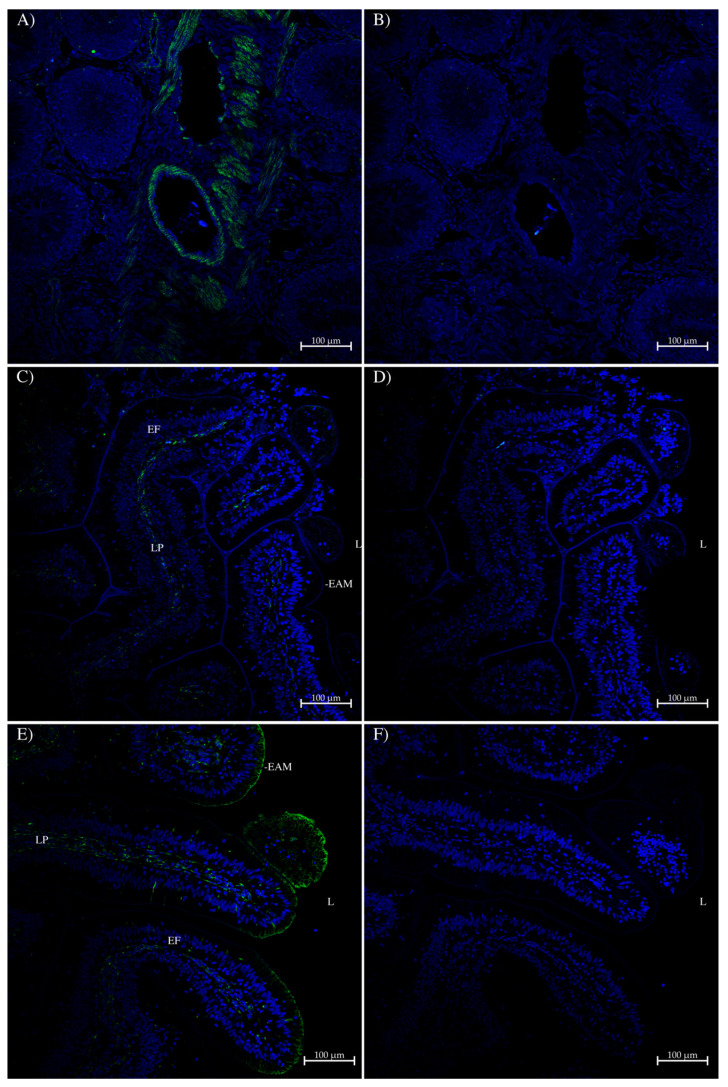
Immunohistochemistry of spiny dogfish intestinal spiral valve flap. Sections were imaged on a Zeiss 710 laser-scanning confocal microscope. All sections were imaged with a 20× lens with a zoom setting of 0.6. In sections (**A**,**C**), AQP1/2 antibodies were stained green with Alexa 488 plus dye-labeled donkey anti-rabbit IgG secondary antibody (Thermofisher). Sections (**B**,**D**) are control serial sections (comparable to (**A**,**C**), respectively) incubated with peptide-antigen-blocked antibody (1 h, 50 μg/mL peptide). Section (**E**) was generated by using a tyramide amplification kit (Thermofisher). Section (**F**) is a control serial section that lacked the primary AQP1/2 antibody. L = intestinal lumen; EF = epithelial fold; LP = lamina propria; and EAM = epithelial apical membrane. All sections were processed with True Black autofluorescence quencher (Biotium), and the blue stain is DAPI nuclear counterstain. Sizes are indicated with scale bars.

The staining in the intestinal spiral valve flap (Figure 9) was very similar to that of the side wall. There was AQP1/2 antibody staining in central muscle blocks and in blood vessels and also staining in the lamina propria of the epithelial folds (Figure 9A,C). Control serial sections incubated with peptide-antigen-blocked antibody (Figure 9B,D) showed no similar staining. When a tyramide amplification kit was used, there was a moderately strong apical membrane staining of the surface epithelial cells of epithelial folds (Figure 9E), which was not seen on a serial section incubated without the AQP1/2 antibody.

**Figure 10:**
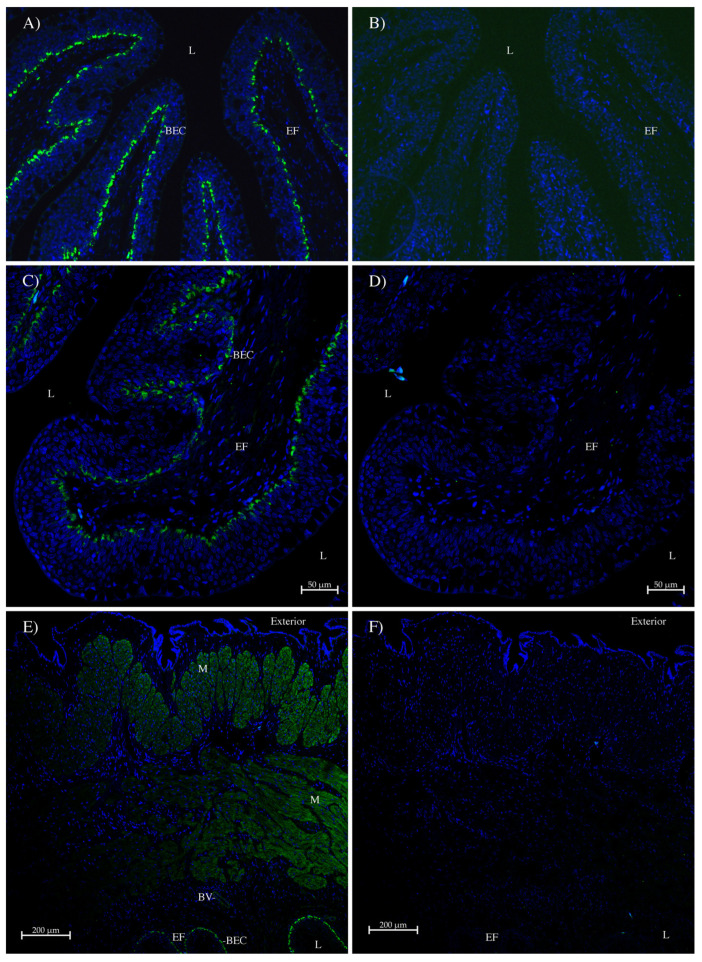
Immunohistochemistry of spiny dogfish rectum/colon. Sections (**A**,**B**) (100× magnification) were imaged with a Zeiss Axiovert microscope. In (**A**), green staining comes from the AQP1 antibody incubated with an Alexa 488 dye-labeled donkey anti-rabbit IgG secondary antibody (Thermofisher). Sections (**B**,**D**,**F**) are control serial sections (comparable to sections (**A**,**C**,**E**), respectively) incubated with peptide-antigen-blocked antibody (1 h, 50 μg/mL peptide). Sections (**C**–**F**) were imaged on a Zeiss 710 laser-scanning confocal microscope, were processed with True Black autofluorescence quencher (Biotium) and have sizes indicated with scale bars. Sections (**C**–**D**) are serial sections that used a 20× lens and a zoom factor of 0.9. Sections (**E**,**F**) are serial sections that used a 10× lens and a zoom factor of 0.6. Sections (**C**,**E**) were incubated with the AQP1/2 antibody and Alexa 488 Plus dye-labeled (green) donkey anti-rabbit IgG secondary antibody (Thermofisher). L = intestinal lumen; EF = epithelial fold; BEC = basal epithelial cell; M = muscle; and BV = blood vessel. The blue stain is DAPI nuclear counterstain.

In the spiny dogfish rectum/colon, the AQP1 antibody stained a layer of cells at the base of the surface epithelium (Figure 10A). A control serial section (Figure 10B), incubated with peptide-antigen-blocked antibody, showed no similar staining. Likewise, sections incubated with the AQP1/2 antibody also stained the same cells at the base of the surface epithelium (Figure 10C), and a control serial section (Figure 10D) incubated with peptide-antigen-blocked antibody showed no similar staining. A wide-field image of the rectal/colon tissue (Figure 10E) additionally showed staining in muscle blocks and blood vessels in the body of the tissue, and again, on a control serial section (Figure 10F) incubated with peptide-antigen-blocked antibody, there was no similar staining.

**Figure 11:**
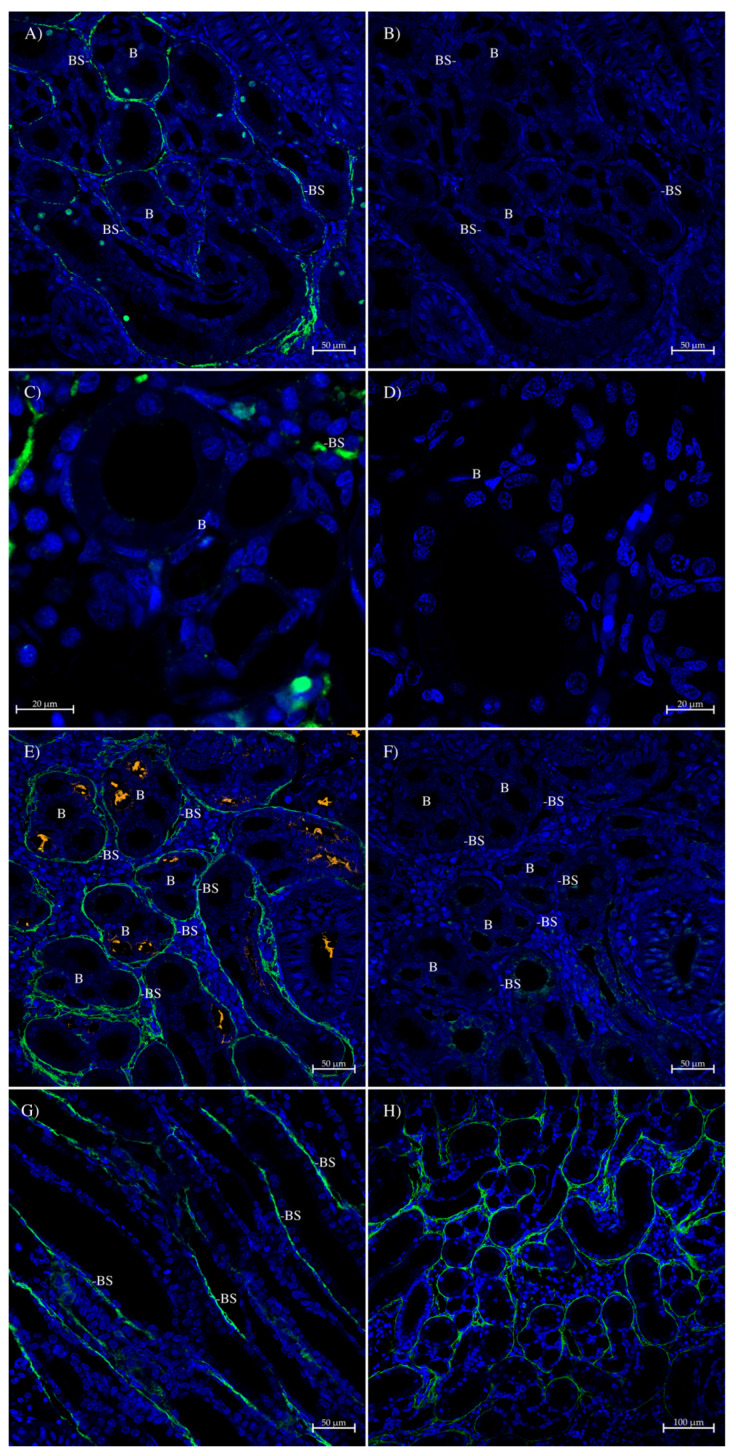
Immunohistochemistry of spiny dogfish kidney bundle zone. All sections were imaged on a Zeiss 710 laser-scanning confocal microscope and were processed with True Black autofluorescence quencher (Biotium) and have sizes indicated with scale bars. Sections (**A**–**G**) were generated by using a Tyramide amplification kit (Thermofisher), which deposits Alexa 488 dye (green) around the location of bound primary antibody. Section (**E**) was additionally incubated with mouse anti-acetylated tubulin antibody (Sigma, St Louis, MO, USA), detected with a highly cross-absorbed goat anti-mouse IgG secondary antibody with Alexa 555 dye attached (orange). The acetylated tubulin antibody labels cilia that are prevalent in the proximal parts of the nephron tubule lumen. Green staining on section (**H**) comes from antibodies incubated with an Alexa 488 dye-labeled donkey anti-rabbit IgG secondary antibody (Thermofisher). Section (**A**) (20× lens, zoom of 1.0) was incubated with the AQP1 antibody and shows bundle sheath (BS) staining in the convoluted bundle zone around nephron tubule bundles. In section (**B**), no similar staining was seen in the control serial section (section (**B**)); 20× lens, zoom of 1.0)) incubated with peptide-antigen-blocked antibody (1 h, 50 μg/mL peptide). Section (**C**) (40× lens, zoom of 1.7) shows the AQP1SV1 antibody staining of the bundle sheath in the convoluted bundle zone in comparison with a section (section (**D**); 40× lens, zoom of 1.4) incubated with no primary antibody. Section (**E**) (20× lens, zoom of 1.0) was incubated with the AQP1/2 antibody and shows bundle sheath staining in the convoluted bundle zone around nephron tubule bundles (labeled with a ‘B’). No similar staining was seen on the control serial section (section (**F**)); 20× lens, zoom of 1.0) incubated with peptide-antigen-blocked antibody. Section (**G**) (20× lens, zoom of 1.0) shows the AQP1/2 antibody staining of the bundle sheath in the straight bundle zone. Section (**H**) (20× lens, zoom of 0.6) is a wide-field image of the convoluted bundle zone with AQP1/2 antibody staining the bundle sheath. The blue stain is DAPI nuclear counterstain. All sections were processed with True Black autofluorescence quencher (Biotium).

In the spiny dogfish kidney bundle zone, all three antibodies stained the bundle sheath that surrounds nephron bundles. This was true for the AQP1 antibody (Figure 11A) in the convoluted bundle zone, compared with a control serial section (Figure 11B) incubated with peptide-antigen-blocked antibody that showed no similar staining. Although the staining was weaker and patchy, it was also true for the AQP1SV1 antibody in the convoluted bundle zone (Figure 11C) in comparison with a section incubated with no antibody (Figure 11D). The convoluted bundle zone bundles (of cross-sections of five tubules) were most clearly surrounded by bundle sheath staining in the image that was generated with AQP1/2 antibody (Figure 11E) in comparison with its control serial section (Figure 11F), where the antibody was incubated with peptide-antigen-blocked antibody. A wide-field image of AQP1/2 antibody bundle sheath staining was also obtained (Figure 11H). The bundle sheath staining with the AQP1/2 antibody extends into the straight part of the bundle zone (Figure 11G), which runs from the lateral sides of the kidney towards the center at periodic intervals along the kidney’s posterior-to-anterior length.

**Figure 12:**
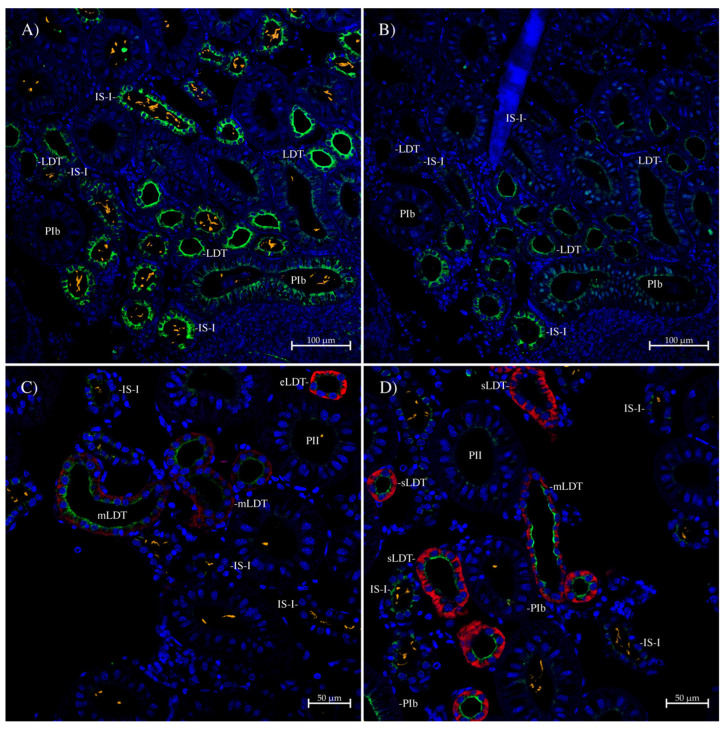
Immunohistochemistry of spiny dogfish kidney sinus zone. All sections were imaged on a Zeiss 710 laser-scanning confocal microscope and were generated using a Tyramide amplification kit (Thermofisher), which deposits Alexa 488 dye (green) around the location of the bound primary antibody. Sections were also processed with True Black autofluorescence quencher (Biotium) and have sizes indicated with scale bars. In section (**A**) (20× lens, zoom of 0.7), there was additional incubation with mouse anti-acetylated tubulin antibody (Sigma), detected with highly cross-absorbed goat anti-mouse IgG secondary antibody with Alexa 555 dye attached (orange). The AQP1/2 antibody strongly labeled the apical pole/membranes of LDTs (no cilia) with slightly less strong apical pole staining in IS-I tubule segments and lower-level apical pole staining in some PIb/PII tubules. This staining was partially blocked out in a control serial section (section (**B**)); 20× lens, zoom of 0.7) incubated with peptide-antigen-blocked antibody (1 h, 50 μg/mL peptide). Section (**C**) (20× lens, zoom of 1.0) was additionally stained with rabbit anti-spiny dogfish AQP3 antibody directly labeled with a CF633 dye mix-n-stain antibody labeling kit (Biotium; red). The AQP3 antibody shows increased staining along the length of the LDT from the early part of the middle LDT onwards [26]. Section (**D**) (20× lens, zoom of 1.0) was also additionally stained with rabbit anti-spiny dogfish AQP4/2 antibody directly labeled with CF633 dye (Biotium; red). AQP4/2 labels the start of the LDT strongly, with decreased staining further along the LDT [26]. AQP1/2 antibody staining (green) was the strongest in the middle LDT (mLDT) and some parts of the start of the LDT (sLDT). eLDT = end of the LDT. The blue stain is DAPI nuclear counterstain. All sections were processed with True Black autofluorescence quencher (Biotium).

In the spiny dogfish kidney sinus zone, the tyramide-amplified staining with the AQP1/2 antibody was strong (Figure 12A), and it was hard to block it with the peptide antigen, although a partial block was achieved (Figure 12B). The AQP1/2 antibody staining was seen in some nephrons but not others and was strongly located in the apical pole and apical membrane of LDT tubules (as indicated by the lack of cilia), with somewhat less intense staining in the IS-I segment tubules (as indicated by abundant cilia {orange staining}). There was also some weaker staining in the apical pole of some larger PIb and/or PII tubules. The spiny dogfish AQP3 and AQP4/2 antibodies (see [26]) were also used to identify the parts of the LDT that the AQP1/2 antibody stained (Figure 12C and Figure 12D, respectively). The AQP1 staining was variable but appeared to be strongest in the middle of the LDT (mLDT) and some parts at the start of the LDT (sLDT; where AQP4/2 staining is strong), with lower levels where AQP3 staining is strong towards the end of the LDT (eLDT).

## 3. Discussion

The mRNA expression profiles of *AQP1* and *AQP1SV1* were fairly similar, with very high levels of expression in the rectal gland and with lower levels in other tissues (Figure 2A). The more exhaustive PCR showed that there was some expression in all the tissues studied (Figure 2B). This might indicate expression in blood cells as it occurs in mammals [3], as every tissue has blood in it. However, the staining with the AQP1/2 antibody in the gill (Figure 7E) suggested that red blood cells in the spiny dogfish, at least, do not appear to express AQP1. The level of mRNA in the rectal gland was very high, around 6.5× that of *AQP4* according to Northern blotting (these blots were produced at the same time and in the same way) [39]. Northern blotting showed there were significantly lower levels of *AQP1* mRNA in the rectal gland of 120% SW-acclimated fish (Figure 3A). The expression of the splice variant *AQP1SV1* showed a similar rectal gland expression profile to that of *AQP1*, although the significant differences were higher levels of mRNA expression in the 75% SW fish compared with the other two groups (Figure 3B). These differences in rectal gland *AQP1*/*AQP1SV1* mRNA expression were in contrast to the level of *AQP4* mRNA levels in the same samples, where there were no differences in *AQP4* mRNA expression among fish acclimated to the different salinities [39]. The lower level of *AQP1* expression in 120% SW fish makes logical sense, in that in elevated environmental salinities above the normal plasma concentration of the spiny dogfish, fish would start to dehydrate due to water losses across the gill and with losses due to urine production. Consequently, it would be beneficial for the animals not to lose more water due to rectal gland secretions. Probably, a tendency towards decreasing blood pressure due to dehydration would cause a regulated shutdown of the gland, although this could also be achieved by reducing blood flow to the gland [41,42], as well as reducing fluid secretions by reducing the level of AQP1 expression. Rectal gland secretions are known to be under hormonal control [23,42,43,44,45,46,47]. The level of AQP4 in rectal gland secretory tubule basolateral membranes would be less important, as without an exit pathway for water into the tubule lumen through apical AQP1, AQP4′s role (in the fluid secretion mechanism) would be naturally curtailed anyway. The changes in *AQP1* mRNA expression in kidney and intestine also make sense, in that the 120% SW dogfish had significantly higher *AQP1* mRNA levels than the 100% SW fish. Again, in 120% SW, dogfish would try to absorb/reabsorb more water both from the intestine and in the kidney, and higher levels of AQP1 would facilitate that. The levels of *AQP1SV1* mRNA in kidney and intestine showed the same trends as *AQP1* mRNA (higher in 120% SW fish), but the differences were not statistically significant. Also, in the gill, it might be expected that *AQP1* mRNA levels would be lower in 120% SW fish, and *AQP1SV1* mRNA levels showed that trend without being statistically significantly different from 100% SW fish.

One of the aspects that was most curious in this study was that the level of AQP1 protein (staining) in the rectal gland seemed to be very low in terms of AQP1 antibody staining, even with AQP1/2 antibody staining (considering the up to 200-fold amplification with the tyramide kit used), and that is in comparison to easily detectable rectal gland AQP4 staining [36]. The distribution of staining in the gland was also different between AQP1 and AQP4 (Figure 6 and [36]). AQP4 staining was fairly ubiquitous within the tubules of the gland, whereas AQP1 staining was mainly located in tubules located towards the periphery of the gland. A lower level of apical staining was also seen in tubules especially close to the rectal gland duct. The reason for this is not apparent, although it suggests that the tubules near the periphery are more actively secreting fluid.

In the gill, the staining with AQP1 and AQP1SV1 antibodies looked largely like autofluorescence in red blood cells (these images were taken before the advent of true black autofluoresence quencher), although there may be small amounts of staining in the basal membranes of secondary-lamellar pavement cells. That staining showed up strongly with the much stronger AQP1/2 antibody and is absent in the peptide-blocked-antibody controls (Figure 7C–F). The presence of an aquaporin on the basal membrane of secondary-lamellar pavement cells is much less problematic than it potentially would be, for example, for a teleost fish, due to the smaller osmotic differences between the fish’s plasma and the SW environment in spiny dogfish (in comparison with teleosts) [11,14,15,16,17]. The presence of AQP1 on the basal membrane of secondary-lamellar pavement cells suggests that these cells may have issues with influxes of water into the cell cytoplasm from the external environment that would require an efflux pathway out the cell that was faster than diffusion through the plasma membrane. Any influxes occurring could be alleviated by AQP1 allowing water to flow out of these cells and into body fluids across the basal cell membranes. The AQP1/2 antibody also showed some staining around blood vessels in the gill arch. This may be to facilitate the movement of any absorbed water into the bloodstream in other parts of the gill.

The AQP1/2 antibody staining in the spiral valve intestinal side wall and spiral valve flap were fairly similar, with staining in muscle tissue (similar to AQP4; [10]) and the surrounding blood vessels and in the lamina propria connective tissue in the middle of luminal surface epithelial folds (Figure 8 and Figure 9A–D). AQP1 is known to be expressed in mammalian muscle [1], but one always has to be circumspect about muscle and blood vessel staining due to the presence of autofluorescence in those tissues. However, the staining appears specific to the antibody in comparison with control section images taken by using the same microscope settings. Because the RNA samples from the intestine were produced from epithelial tissue scrapes, the relatively high levels of AQP1 expression seen in underlying smooth muscle in the immunohistochemistry would not have been present in the RNA samples used to make cDNA and hence would not have been reflected in the tissue PCR shown in Figure 2. With tyramide amplification, a low level of staining was detected for the AQP1/2 antibody in the apical membranes of surface epithelial cells towards the end of the epithelial folds, i.e., in the cells most in contact with luminal fluids (Figure 8 and Figure 9E,F). These cells are also thought to express AQP3 on their basal membranes and AQP15 on the apical and lateral membranes [38]. In the colon/rectum (Figure 10), the staining was a little different from the spiral valve intestine. There was the same staining in the muscle and surrounding blood vessels as in the intestine, but there was also intense staining in a layer of cells at the base of the surface epithelium. This was strong enough that both the AQP1 (Figure 10A,B) and AQP1/2 (Figure 10C–F) antibodies detected it. The staining was absent in peptide-antigen-blocked antibody control serial sections. The reason for AQP1 expression in this particular location is not clear, although it may just concern the need to control cell volume in these cells.

In the kidney bundle zone, the AQP1, AQP1/2 and to a lesser extent the AQP1SV1 antibodies all stained the peritubular bundle sheath cells (Figure 11). This shows similar localization to that of the AQP15 antibody, but staining was not seen in this location with the AQP4 or AQP3/2 antibodies, which instead stained the basal membrane of the EDT tubules, or the AQP3 antibody, which shows no staining in the bundle zone [26,37]. The peritubular bundle sheath surrounds the five tubules in bundles (seen in cross–section in the convoluted bundle zone), which are thought to be involved in reabsorption using a counter-current mechanism [27,28]. The staining in the peritubular bundle sheath with both AQP1 and AQP15, but not other AQPs, makes sense on one level, in that they are evolutionarily closely related, with both coming from an ancient duplication event [4]. As noted previously [26], the expression of both AQP1 and AQP15 in the bundle sheath (in both the convoluted and straight bundle zone regions) is probably there to provide an entry route for water into the bundle to allow for the creation of fluid for solutes reabsorbed from the nephron tubules. The fluid may either drain out directly from the bundles into the sinus zone or exit via a central vessel in the bundle, or it may be picked up by blood vessels that occur in the bundle zone [31,48]. In the sinus zone, the AQP1/2 antibody stained strongly the apical membranes of LDT tubules (Figure 12A), which lack luminal cilia. The staining was further localized with AQP3 and AQP4/2 antibodies that stain the start (AQP4/2) and end (AQP3) of the LDT segment strongly [38]. The AQP1 staining in this experiment was lower and more patchy, with AQP1 staining predominantly in the middle part of the LDT segment loop and in some parts at the start of the LDT, where AQP4/2 staining was strong (Figure 12C,D). There was also apical membrane/pole staining, at a slightly lower but still strong level, in IS-I tubule segments which are located at the end of the first sinus zone nephron loop. Additionally, there was some lower-level PIb and PII tubule segment apical pole staining (start and middle of the first sinus zone loop). The staining in the IS-I, PIb and PII tubules was inconsistent, with some tubules having staining and others apparently having less or none. This could just mean that AQP1 was only expressed in parts of these tubule segments, but it may also mean that only certain nephrons were expressing AQP1. This may fit in with the fact that only certain nephrons are thought to be actively filtering and receiving glomerular filtrate [33,49]. It is speculation, but it might be that non-filtering nephrons have lower or no AQP1 expression.

For the Western blotting, there appeared to be four bands on the blot with the AQP1SV1 antibody, one of which was at the expected size of AQP1SV1, as well as three low-molecular-weight bands. What the three larger bands and the three low-molecular-weight bands represent is unknown. The AQP1SV1 antibody only remained functional for a short while, and before very much could be done with it, it became non-functional. The AQP1 antibody was somewhat better and, with an extremely long substrate exposure, gave a band of the expected size of 28 kDa on its Western blot. The AQP1/2 antibody gave a similarly sized band and two others around 31 and 32 kDa, which may represent larger versions of the protein, such as what might be produced by further splice variants. Alternatively, the two bands might be glycosylated forms of AQP1. There were also two bands at around 52–54 kDa, which may be dimers of either AQP1-AQP1SV1 or AQP1-AQP1. The band around 250 kDa could be some kind of larger AQP1 multimer but may also be an aggregate of some kind that includes AQP1 proteins. Of course there is also always the possibility that the additional bands might just be due to the non-specific binding of the antibody. Polyclonal antibodies such as those used in this study rarely give the perfect/expected result. While they are relatively specific and appear to work well most of the time, there is always the possibility of cross-reactivity to other similar proteins. That is probably truer in Western blotting than in immunohistochemistry but is always a possibility with either technique. Still, it is far better to have some reasonably reliable localization information that can be cross-checked later using other approaches than to know nothing at all.

The functional expression data showed that AQP1 is a water channel and has the characteristics that might be expected of a member of the water-selective aquaporin sub-group. What was interesting was that AQP1SV1 with just a different and truncated C-terminal end with no cytoplasmic tail appeared to be non-functional in terms of its water permeability characteristics. This was unexpected, as a Human AQP1 mutant generated without a C-terminal tail had normal water permeability characteristics, although the missing region interfered with the ability of the cells expressing the AQP1 mutant to migrate or proliferate normally [50]. The lack of functional water permeability of AQP1SV1 suggests that it may play a different role, which might be some kind of regulator that interferes with the function of AQP1 expression or activity either at the protein or mRNA level or both. An attempt was made to show AQP1SV1 expression on the membrane of oocytes by labeling the c-myc tag with mouse anti-c-myc primary antibody and Alexa488-labeled anti-mouse IgG antibody however, the background fluorescence in the control oocytes was too high for this to be conclusive. Other (now eight) aquaporin transcripts that have been expressed were all functional, suggesting that the technique normally results in the delivery of expressed proteins to the oocyte membrane. Consequently, it seems much more likely than not that AQP1SV1 was also being delivered to the oocyte membrane.

The C-terminal domain of AQP1 is known to be involved in the regulation of AQP1 [51,52]. In mammals, tetramers of AQP1 are thought to form an ion channel through the central channel between the four AQP1 monomers, and this ion channel activity is gated by cGMP [51,52,53,54]. Mutation in the tyrosine residue (located at position 249 in the C-terminal tail of the spiny dogfish AQP1 sequence) disrupted cGMP gating [51,54]. However, this tyrosine of the AQP1 C-terminus has also been suggested to be a calcium-binding protein site [55,56]. Additionally, the amino acid residue at position 235 (serine in the C-terminal tail of spiny dogfish AQP) of the sequence has been implicated in protein kinase C (PKC) phosphorylation and the regulation of Human AQP1, although the residue is a threonine in that sequence [51,56,57]. PKC regulates the trafficking of AQP1 from the plasma membrane in response to hypotonicity [51,56,57,58]. Lastly, AQP1 membrane trafficking is known to also be regulated by protein kinase A (PKA), although the AQP1 site(s) that PKA phosphorylates is unknown [51,57,59]. The lack of the C-terminal cytoplasmic tail in AQP1SV1 suggests that it would also lack much of the likely regulation that may control the complete AQP1 protein itself.

## 4. Methods and Materials

### 4.1. Fish Samples

Samples from the fish in this study have been used many times for studies on aquaporin and other genes, for example, in [8,9,26,35,36,37,38]. As in those studies, IACUC approval was granted on a yearly basis both by the Mount Desert Island Biological Laboratory (MDIBL; e.g., A3562-01; 26 June 2007) and/or Georgia Southern University (e.g., I06050; 27 May 2007). Samples for DNA cloning, tissue PCR, protein blotting and immunohistochemistry were selected randomly from a communal stock tank held in local SW and fed a diet of squid daily. Gill and gastrointestinal tract tissues taken for RNA samples were epithelial scrapes (obtained using a razor blade and a microscope slide, respectively) and hence contain little to no underlying smooth muscle or cartilage. Fish for the salinity acclimation experiment were acclimated in stages to 120 or 75% SW over the course of one week and were unfed after the onset of the experiment. For details of the acclimations, see [39].

### 4.2. PCR, DNA Cloning and Northern Blotting

The original fragment of spiny dogfish *AQP1* was amplified using a degenerate PCR approach as used many times before (e.g., see [38,39]). This uses two regions of evolutionarily conserved amino acids in protein sequences reverse-translated into nucleotides and uses the universal base, inosine, at positions of significant sequence uncertainty (see Table 1). The ends of the *AQP1* cDNA sequence were then amplified using RACE PCR and a Marathon cDNA synthesis kit (Originally Clontech, now Takara, San Jose, CA, USA). During the 3′RACE experiments, 4 different 3′ end sequences were obtained, and the first two are here referred to as *AQP1* and *AQP1SV1* (Accession Numbers PV254899 and PV254900, respectively). The other two sequences would not amplify from cDNA and were deemed to be artifacts of the 3′RACE ligation step. Tissue PCR (using cDNAs from gill, esophagus/fundic stomach, rectal gland, kidney, intestine, stomach, brain, skeletal muscle, eye and liver) was carried out in two different ways to determine the tissue distribution of the expression of both *AQP1* and *AQP1SV1*. Stock cDNAs (PCR using cDNAs made with the same volume of total RNA but with variable RNA amounts) were used to see the approximate distribution of expression. These PCR reactions amplified the whole coding region (this amplificon runs across 4 exons, and the product would be significantly larger than expected if genomic DNA had amplified), using a limited number of 30 PCR cycles, and used Taq DNA polymerase (NEB, Ipswich, MA, USA) and 1/40th of the cDNA reaction (0.5 μL out of 20 μL). Secondly, more exhaustive PCR amplifications were performed to check for expression in tissues with lower amounts of AQP1. These reactions used cDNA made with 1 µg of total RNA each, amplifying a short DNA fragment, using more (35) PCR cycles, more efficient Phusion DNA polymerase (NEB) and 1/200th of the cDNA reaction (1 μL out of 20 μL diluted to 200 μL). For these amplifications, the sense primers were made across the intron–exon junction region where the AQP1 and AQP1SV1 sequences diverge, and the antisense primers were in the two different 3′ end sequences. Quantitative PCR (QPCR) was carried out in select tissue cDNAs by using an MX4000 QPCR machine and Brilliant II QPCR mastermix (Stratagene, La Jolla, CA, USA), as in [39]. Northern blotting was also carried out for *AQP1* mRNAs, and these Northern blots were quantified by Instant Imager (Canberra Packard, Schwadorf, Austria), as in [39]. For both Northen blotting and QPCR, the total RNA samples used to make cDNAs were normalized using 18S rRNA levels on agarose electrophoresis gels. Northern blots were carried out by using fragments generated with the short or QPCR primers (see Table 1). The size of *AQP1* mRNAs was determined by using RNA markers (Gibco/BRL, now Life Technologies, Paisley, Scotland) on agarose electrophoresis gel. The statistical testing of results was performed using Statsview software (Abacus concepts, Mindvision software, Version 4.01, Adelaide, Australia), using ANOVA with Fisher’s PLSD post hoc test of significance. 

### 4.3. Functional Studies

The functional studies were carried as in [39] using c-myc-tagged AQP sequences. Indeed, as the oocytes used in this study were performed in parallel with those in the study cited, the control oocytes are essentially the same. The data were processed slightly differently (see below) with the radius of the projected oocyte image being back-calculated from the area; then, this radius was used to calculate the volume of a sphere (4/3.pi.r^3^). Data were then normalized and processed as in [39].

#### 4.3.1. Production of Aquaporin cRNAs for Micro-Injection

Aquaporin sequences were c-myc-tagged at the C-terminal end and sequenced. Plasmid DNA for *AQP1* and *AQP1SV1* was cut with restriction endonuclease XbaI (New England Biolabs, Beverly, MA, USA) to linearize and prevent the run-on of transcription. cRNA transcripts were produced using an mMessage mMachine kit (Ambion, Austin, TX, USA) utilizing T7 DNA-dependent RNA Polymerase. The cRNA produced was purified using phenol/chloroform extraction followed by isopropanol precipitation, dissolved in RNase-free water and quantified by using a Biophotometer spectrophotometer (Eppendorf, Westbury, NY, USA).

#### 4.3.2. Preparation of *Xenopus laevis* Oocytes

All experiments were performed in accordance with IACUC-approved protocols at Beth Israel Deaconess Medical Center. Stage V–VI oocytes were harvested from female Xenopus laevis, and cRNA was prepared and injected into oocytes as described in MacIver et al. [60]. *Xenopus laevis* frogs (Harvard Institute of Medicine, Boston, MA, USA) were anesthetized in 1 L of 0.5% (*w*/*v*) 3-aminobenzoic acid ethyl ester methanesulfonate salt (Tricaine) containing ice for 20 min. Oocytes were removed bilaterally from the abdominal cavity, and the egg mass was cut into small pieces and placed in calcium-free ND96 buffer (in mM: 96 NaCl, 1 KCl and 1 MgCl_2_ 5 Hepes, pH 7.5). Oocytes were then defolliculated in 2 mg/mL collagenase (Sigma-Aldrich, St Louis, MO, USA) and 0.2 mg/mL trypsin inhibitor (Sigma-Aldrich) in calcium-free ND96 for 55 min with rotation on an Adams Nutator before being washed three times with phosphate buffer (in mM: 100 K_2_HPO_4_ and 0.1% (*w*/*v*) BSA, pH 6.5); then, oocytes were allowed to incubate in phosphate buffer for 10 min at room temperature. Oocytes were transferred to calcium-free ND96 and then to modified Barth’s solution (MBS; in mM: 88 NaCl, 1 KCl, 2.4 NaHCO_3_, 0.82 MgSO_4_, 0.33 Ca(NO_3_)_2_, 0.41 CaCl_2_ and 10 Hepes, pH 7.4, supplemented with 1% *v*/*v* penicillin/streptomycin), where they were maintained at 18 °C.

The cRNA (10 ng) of *AQP1* or *AQP1SV1* was injected into oocytes using a Nanoject II Auto-Nanoliter Injector (Drummond Scientific Co., Broomall, PA, USA). Control oocytes were either injected with water alone or were uninjected. After 3 days of incubation at 18 °C, oocytes were tested for their ability to transport water, urea or glycerol. Water transport kinetics were assessed at room temperature by the quantitation of oocyte swelling after placement in hypotonic buffer (65% of normal modified Barth’s saline—MBS). Time-lapse video microscopy was used to capture oocyte images every 10 s for 3 min using an Olympus SZX7 binocular microscope (Tokyo, Japan) equipped with a Scion CFW 1308C digital camera (1360 × 1024 pixel resolution).

Aquaporin activity was tested over a pH range of 6.6 to 8.6. For all experiments other than pH 7.4, oocytes were placed in MBS at the tested pH for 5 min and then were allowed to swell in hypotonic MBS (65%) at the same pH.

#### 4.3.3. Calculation of Permeability Coefficients

The images were converted into black and white in ImageJ (Rasband, W.S., ImageJ, U. S. National Institutes of Health, Bethesda, MD, USA, http://imagej.nih.gov/ij/, versions 0.5–1.45s, 1997–2011) by using the Binary function, and the cross-sectional pixel area was calculated with the Analyze Particle function of the expanding circle. The data from ImageJ were exported to Microsoft Excel. The radius of the expanding circle was calculated by using the area of a circle = pi x radius^2^. The radius was then used to calculate the volume of the oocyte assuming a uniform sphere (4/3 pi r^3^). The volume was then normalized to 1.0 relative to the starting value. To calculate the apparent permeability coefficient (P_f_), the data were plotted as time vs. relative volume; then, a second-order polynomial equation was fitted, and the derivative of the equation was used to obtain the initial rate of swelling. P_f_ was calculated using the following equation [61]:

$$P_{f}=d(V/Vo)/dt\;\times \;Vo\;\times \;(1/S)\;\times \;(1/Vw)\;\times \;(1/\underset{=}{∆}C)$$

where d(V/Vo)/dt is the rate from the curve fit; Vo is the initial volume of the oocyte, calculated as 5.2 × 10^−4^ cm^3^ based on a 1 mm diameter; S is the surface area of the oocyte (0.4 cm^2^; [62]); Vw is the molar volume of water (18 mol/cm^3^); and ∆C is the concentration difference of the applied hypo-osmotic solution in mol/cm^3^. Statistical testing used Student *t*-tests in Microsoft Excel.

#### 4.3.4. Urea and Glycerol Uptake

Solute fluxes were measured by the isotopic uptake of [^3^H]glycerol or [^14^C]urea (American Radiolabelled Chemicals, St. Louis, MO, USA), using a similar methodology to that in [60]. Oocytes (8–12) were incubated at room temperature for 90 s in MBS which included either 10 μCi/mL [^14^C]urea (55 mCi/mmol) or 10 μCi/mL [^3^H]glycerol (20–40 Ci/mmol), made to a final concentration of 2 mM with unlabeled urea or glycerol, respectively. At the end of the incubation period for uptake, oocytes were washed six times with ice-cold buffer containing 5 mM solute. Individual oocytes were then placed in scintillation vials, had 300 μL of 20% SDS added and were vortex-mixed for 10–15s before the addition of 4 mL of Scintisafe scintillation cocktail (Fisher Science, Waltham, MA, USA). Vials were counted for 2 min in a Packard 2200CA liquid scintillation analyzer (Perkin Elmer, Shelton, CT, USA). Statistical testing used Student *t*-tests in Microsoft Excel.

### 4.4. Antibodies, Western Blotting and Immunohistochemistry

For initial studies, two polyclonal antibodies were made by Prosci Inc. (Poway, CA, USA) against the C-terminal sequences of AQP1 (NH2- CGGYDVEGEGDSARMEMKPK -COOH; located at amino acids 249–265 of the alignment in Figure 1) and AQP1SV1 (NH2- CGGGERQRAPWISSQYSIG -COOH; located at amino acids 213–227 of the alignment in Figure 1). An amino-terminal cysteine was added to the peptide for attachment to the carrier protein (keyhole limpet hemocyanin {KLH}) and affinity purification media, and low-antigenicity glycines (with only a hydrogen side chain group) were added as spacers to alleviate steric hinderance near the carrier protein’s surface. Both of these antibodies turned out to give weak signals in Western blotting and immunohistochemistry, so another AQP1 antibody was subsequently made (AQP1/2) by Genscript (Piscataway, NJ, USA) by using a peptide sequence (NH2- CGNSGQTEEYDVEGEGDSAR -COOH; located at amino acids 242–259 of the alignment in Figure 1) from slightly further in from the end of the carboxyl (C)-terminus of the AQP1 protein (see Figure 1). There may have been some issues with the solubility of the AQP1/2 peptide antigen, as control experiments using the peptide blocking of antibodies were highly variable in their results (no blocking to complete blocking), so controls were sometimes performed without the primary antibody instead of the use of peptide antigen blocking. Tissue fixation, sectioning and immunohistochemistry were carried out as in [38,40].

Western blotting was carried out in a similar way to previously [26,35,36,37], using 300 μg of either rectal gland crude membrane extracts or kidney purified plasma membranes. Likewise, tissue fixation, sectioning and immunohistochemistry were carried out using a methodology similar to that in [26,35,37,38]. Details for individual images vary and so are found in the respective figure legends (for Figure 6, Figure 7, Figure 8, Figure 9, Figure 10, Figure 11 and Figure 12).

### 4.5. Statistical Testing

For the environmental salinity experimental data (Figure 3), ANOVA statistical tests were performed with Fisher’s PLSD post hoc test of significance between groups (using Statsview statistical software, Abacus Concepts, Mindvision software, version 4.01, Adelaide, Australia), where there were *n* = 6 fish (tissue total RNA samples) per group. For the functional expression experiments (Figure 4), unpaired *t*-tests were used for statistical comparisons (using the Microsoft Excel T-test function). The normality of the data was not tested due to the small sample sizes involved. In all statistical tests, significance was determined as the probability (*p*) of groups being different due to random chance as * *p* < 0.05, ** *p* < 0.01, *** *p* < 0.001 and **** *p* < 0.0001.

## 5. Conclusions

Numerous studies were conducted over a period of 21 years from 2004 to 2025 to make this article as comprehensive as possible given the very limited amount of resources available. As in other organisms such as mammals, aquaporin 1 appears to be a ubiquitously expressed gene/protein found at some level in a wide variety of tissues. It appears that its most dominant role is in the rectal gland and its likely involvement in rectal gland fluid secretions. However, even with those results, there were some surprises with the level of AQP1 protein being apparently limited in amount in comparison with the relatively high level of *AQP1* mRNA present. Of course AQP1 appears to be playing a number of roles in other tissues, including, putatively, gill pavement-cell volume control, concerning likely water influxes across the gill epithelium; fluid production in the bundle zone of the kidney; facilitating water flows across the peritubular bundle sheath; water reabsorption from forming urine in the sinus zone nephron loops; finally, a probably minor role in fluid absorption in the intestinal tract. Additionally, the apparent splice variant, AQP1SV1, showed no apparent water permeability when expressed in *Xenopus* oocytes, which leads to questions and speculation about its role, which possibly concerns some form of inhibitory regulation due to interference with AQP1 function or *AQP1* expression.

## Figures and Tables

**Table 1:** PCR primers used.

Degenerate PCR primers R = A/G, Y = C/T, K = G/T, I = deoxy-inosine	
Elas *AQP 1e* sen 2	AGY GGI GCI CAR YTI AAY CCI GCI GT
Elas *AQP 1e* anti 1	GA ICK IGC IGG RTT CAT RCT RCA ICC IGT
5′ (5R) and 3′ (3R) RACE primers	
SQ1e 5R anti 1	CCTTCTCCCA ACGCGTTGAC
SQ1e 5R anti 2	CGGAGAGATC CGTCCTTCG
SQ1e 3R sen 1	CGAAGGACGG ATCTCTCCG
SQ1e 3R sen 2	GTCAACGCGT TGGGAGAAGG
Full-coding-region amplifications, etc.	
Squalus *AQP1* sense	TA**GGTACC**AT GATGGTCAGA GAAGTCCAGC GCA
Squalus *AQP1* anti	AT**ACTAGT**TA CTTTGGCTTC ATTTCCATCC TGGC
Squalus *AQP1SV1* anti	AT**ACTAGT**CC CAGTGGCAGA CTCTACCCAA TAGAG
Short and QPCR fragment amplifications (fragment size)	
*AQP1* sense (183bp)	GGA TCA CTG GCT GTA CTG GGT
*AQP1* antisense	GGC TTC ATT TCC ATC CTG GCA C
*AQP1SV1* sense (238bp)	AGG ATC ACT GGG TGA GTT GGT G
*AQP1SV1* anti	CGT GTC CTA ACT CGC ACC ATG

Bolding indicates added restriction enzyme site sequences.

## Data Availability

The authors will make data available to anyone upon request.
